# T cell receptor signaling for γδT cell development

**DOI:** 10.1186/s41232-019-0095-z

**Published:** 2019-03-28

**Authors:** Ryunosuke Muro, Hiroshi Takayanagi, Takeshi Nitta

**Affiliations:** Department of Immunology, Graduate School of Medicine and Faculty of Medicine, The University of Tokyo, Tokyo, 113-0033 Japan

**Keywords:** γδT cell, Thymus, TCR signal

## Abstract

T cells are central to the vertebrate immune system. Two distinct types of T cells, αβT and γδT cells, express different types of T cell antigen receptors (TCRs), αβTCR and γδTCR, respectively, that are composed of different sets of somatically rearranged TCR chains and CD3 subunits. γδT cells have recently attracted considerable attention due to their ability to produce abundant cytokines and versatile roles in host defense, tissue regeneration, inflammation, and autoimmune diseases. Both αβT and γδT cells develop in the thymus. Unlike the development of αβT cells, which depends on αβTCR-mediated positive and negative selection, the development of γδT cells, including the requirement of γδTCR, has been less well understood. αβT cells differentiate into effector cells in the peripheral tissues, whereas γδT cells acquire effector functions during their development in the thymus. In this review, we will discuss the current state of knowledge of the molecular mechanism of TCR signal transduction and its role in the thymic development of γδT cells, particularly highlighting a newly discovered mechanism that controls proinflammatory γδT cell development.

## Background

The immune system of the jawed vertebrates relies on T lymphocytes (T cells) that develop in the thymus. T cells are classified into two types, αβT cells and γδT cells [1]. These different T cell lineages express different types of T cell antigen receptors (TCRs), i.e., αβTCR or γδTCR, that are composed of different sets of somatically rearranged TCR chains and CD3 subunits.

The development and function of αβT cells depend on the αβTCR recognition of antigen peptides presented by the major histocompatibility complex (MHC) proteins. Upon the recognition of the peptide-MHC (pMHC) complex, αβT cells differentiate into effector cells that exert cytotoxic activity or produce cytokines so as to activate innate immune cells or B cells, thus protecting against invading pathogens and tumors [2]. In contrast, no coherent mechanism exists for antigen recognition by γδT cells. The γδTCR reportedly recognizes structurally diverse and biologically unrelated compounds such as lipopeptides, microorganism-derived proteins, and self-proteins. The self-proteins include stress-associated proteins and non-classical MHC [3, 4] as well as classical pMHC complexes [5]. Thus, the antigen recognition mode and differentiation requirements of γδT cells are different from those of αβT cells.

In certain infections, γδT cells, which have the inherent ability to produce cytokines such as interferon-γ (IFNγ) and interleukin-17 (IL-17), contribute to rapid immune responses against a broad spectrum of pathogens and also the smooth transition from the innate to adaptive immune response [4, 6]. Recent studies have demonstrated that IL-17-producing γδT (γδT17) cells have an anti-bacterial ability, but also homeostatic capacity under certain physiological conditions. In the bone fracture repair process, γδT17 cells promote bone regeneration by accelerating osteoblast differentiation [7]. A recent study showed that γδT17 cells in adipose tissue control thermogenesis in response to cold temperature [8]. However, γδT17 cells are also notorious for their ability to induce inflammatory diseases, autoimmunity, and metastasis in mice and humans [9–12]. In particular, γδT17 cells have been reported to play a central role in the pathogenesis of psoriasis, in which IL-17 secreted by γδT17 cells in the skin promotes keratinocyte hyperproliferation and the recruitment of neutrophils [13]. A recent report by Prinz and co-workers demonstrated the non-redundant function of γδT17 cells for psoriasis-like dermatitis using a newly generated mouse strain that enables drug-inducible depletion of γδT cells [14].

Although considerable attention has been paid to the pathophysiological function of proinflammatory γδT cells, it has remained largely unclear how effector γδT cells are generated. Unlike αβT cells, in which effector differentiation occurs in the periphery, both the γδT17- and IFNγ-producing γδT (γδT1) cells are induced during development in the thymus [15]. In the mouse, γδT cells can be sub-classed based on the usage of the TCRγ variable region (Vγ), and the generation of those γδT cell subsets is developmentally regulated during ontogeny: Vγ5 cells develop during the fetal period, Vγ6 cells around birth, Vγ4 cells in the neonatal period, and Vγ1 and Vγ7 cells at adult stage. There is also a close linkage between the Vγ subset and effector function: Vγ4 or Vγ6 cells preferentially include γδT17, while the majority of Vγ1, Vγ5 and Vγ7 cells differentiate into γδT1 [4]. These distinct γδT cell subsets are distributed in lymphoid as well as mucosal tissues.

In this review, we will discuss the current knowledge of the molecular mechanism of γδTCR signal transduction and its role in the thymic development of proinflammatory γδT cells.

## Overview of TCR signaling

**Fig. 1:**
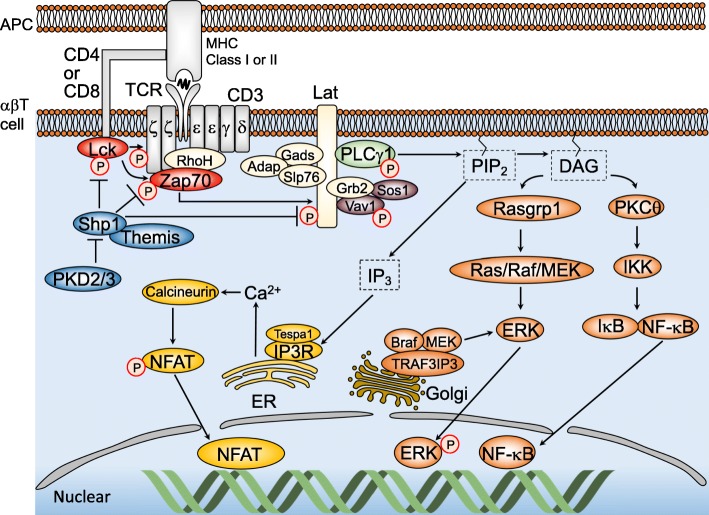
Schematic diagram of αβT cell receptor (TCR) signaling pathway. TCR engagement by pMHC expressed on antigen-presenting cells (APC) induces phosphorylation of CD3 ITAMs by Lck. Zap70 binds to phosphorylated ITAMs and is phosphorylated as well by Lck. The activated Zap70 then phosphorylates Lat, which induces recruitment of adaptor proteins (Gads, Adap, Slp76, and Grb2) and signaling molecules (PLCγ1, Sos1, Vav1). Phospho-PLCγ1 catalyzes hydrolysis of PIP_2_, resulting in generation of DAG and IP_3_. DAG leads to translocation of Rasgrp1 and PKCθ to the plasma membrane, resulting in activation of Ras/MAPK pathway and NF-κB pathway. IP_3_ stimulates endoplasmic reticulum (ER) for the releases of calcium ions, which activate NFAT pathway. Shp1 dephosphorylates a broad range of signaling molecules including Lck, CD3, Zap70, Lat, Slp76, and Vav1, to finely tune TCR signal

The TCR is a complex receptor that consists of receptor subunits (TCRαβ or γδ) and CD3 subunits (CD3γ, δ, ε, and ζ) [16]. TCR signal transduction involves the conformational change, as well as the recruitment and phosphorylation of multiple proteins, including CD3 subunits, kinases, phosphatases, and adaptor proteins (Fig. 1). Among them, most of the kinases act as a driver of TCR signaling. Zap70, a member of the Syk family kinases, plays a key role in TCR signal transduction [17]. In αβT lineage cells, activation of Zap70 is regulated by Lck, a Src family kinase associated with CD4 or CD8 coreceptors. Upon the recognition of pMHC by αβTCR and one of the coreceptors, Lck phosphorylates immunoreceptor tyrosine-based activation motif (ITAM) in CD3 molecules, which induces a recruitment of Zap70 to the αβTCR-CD3 complexes and phosphorylation of Zap70 [2]. Lck also recruits the phosphorylated Zap70 to the transmembrane adaptor protein Lat, and promotes its phosphorylation by Zap70 [18]. The phosphorylation of Lat provides direct as well as indirect docking sites for adaptor proteins such as Grb2, Gads, Slp76, and Adap, signaling enzymes such as PLCγ1 and guanine nucleotide exchange factors such as Vav1 and Sos1. Proteomic analysis has identified the multimolecular complex called the “Lat signalosome”, which is composed of over 100 molecules, indicating that Lat forms a structural scaffold for TCR signaling [19]. PLCγ1 hydrolyzes phosphatidylinositol-4,5-bisphosphate (PIP_2_) into inositol-1,4,5-trisphosphate (IP_3_) and diacylglycerol (DAG). The binding of IP_3_ to the IP_3_ receptor (IP_3_R1) expressed on the endoplasmic reticulum (ER) induces the release of calcium ions from the ER, which in turn stimulates the influx of extracellular calcium ions, resulting in calcineurin activation and nuclear translocation of the transcription factor NFAT. DAG is required for the recruitment of Ras guanyl-releasing protein 1 (Rasgrp1) and protein kinase C (PKC) to the plasma membrane for the activation of the Ras-ERK and NF-κB pathway, respectively [2].

Recently, a genome-wide genetic screening investigation reconfirmed the importance of the known signaling factors such as kinases and adaptor proteins in driving TCR signals. This study in addition identified Fam49b, a cytoskeleton remodeling factor, as a negative regulator of TCR signaling [20]. A set of protein phosphatases have also been shown to negatively regulate the protein phosphorylation events in order to fine-tune TCR signal propagation. These protein phosphatases include Shp1 (also known as Ptpn6), which dephosphorylates crucial tyrosine residues of certain key factors such as Lck [21], CD3ζ [22], Zap70 [23], Vav1 [24, 25], Lat [26], and Slp76 [27], thus inhibiting their signaling activity. T cell-specific Shp1 deletion resulted in an activated CD4 T cell phenotype and an increase in IL-4 production [28].

Although most of the mechanisms of αβTCR signaling mentioned above are thought to be shared by the γδTCR, both the components of the TCR-CD3 complex and receptor-proximal signaling are reportedly different between αβT cells and γδT cells [29]. In fact, the CD3δ subunit is not even incorporated into the γδTCR complex and is not required for γδT cell development [30, 31]. In ex vivo intestinal γδT cells and in vitro activated γδT cells, FcRγ is incorporated into the γδTCR-CD3 complex in substitution for the CD3ζ subunit [30]. Vγ6Vδ1 γδT cells display a higher staining intensity with an anti-CD3ε antibody compared with the other γδT cell populations, suggesting a distinct expression and/or conformation pattern of the CD3 subunits in this γδT cell subset [32]. In addition, a subpopulation of γδT cells is detectable in Lck-deficient or Zap70-deficient mice, whereas αβT cells are completely absent in these mice [33–35]. Considering these observations, it is strongly suggested that αβT cells and γδT cells have distinct molecular mechanisms and requirements for TCR signaling during both their differentiation and activation.

## αβTCR signaling in αβT cell development

αβT cells develop through multiple developmental steps in the thymus. The most immature T cell precursor of both the αβT and γδT cell lineages are CD4/CD8 double-negative (DN) thymocytes. During differentiation through the DN1 (CD44^+^CD25^−^), DN2 (CD44^+^CD25^+^), and DN3 (CD44^−^CD25^+^) stages, they undergo rearrangement of the TCRβ, TCRγ, and TCRδ genes. The successfully rearranged TCRβ chain is assembled with the invariant pTα and CD3 subunits so as to form the pre-TCR complex, which signals in a ligand-independent manner to induce commitment to the αβT cell lineage and differentiation of DN3 cells into DN4 (CD44^−^CD25^−^) cells. This process, termed β-selection, serves as a checkpoint to confirm the generation of a functional TCRβ chain [36].

The pre-TCR signal transduction depends on Syk, another Syk family tyrosine kinase [37], rather than Zap70. Mice deficient in Syk display a reduced transition from the DN3 to DN4 stage, while Zap70-deficient mice display normal differentiation at this stage [38]. Importantly, T cell development is completely arrested at the DN3 stage in Zap70/Syk doubly-deficient mice [38, 39]. Thus, Syk and Zap70 play redundant roles in β-selection, while Syk plays the dominant role. Recruitment of Syk and Zap70 to the CD3ζ chain in the pre-TCR complex is mediated by the adaptor protein RhoH [40–43]. The complete inhibition of β-selection was also observed in Lat-deficient mice, indicating that Lat is a critical target of both Zap70 and Syk in pre-TCR signal transduction [44]. The Lat signalosome triggers the activation of certain downstream pathways required for β-selection, including the Ras/MAPK and NF-κB pathways. Another important signaling pathway for β-selection is regulated by phosphoinositide 3-kinase (PI3K). PI3K, activated by pre-TCR and Notch signals, phosphorylates PIP_2_ so as to generate phosphatidylinositol (3,4,5)-trisphosphate (PIP_3_). PIP_3_ in turn recruits the protein kinases Pdk1 and Pkb (also known collectively as Akt) to the plasma membrane and induces their activation. The PIP_3_ level is negatively regulated by a phosphatase, PTEN. The loss of PTEN results in the bypassing of the pre-TCR and Notch signals in DN3 cells in order to induce the differentiation of DP thymocytes. Therefore, the balance between PI3K and PTEN is critical for early T cell development [45].

The DN4 cells that pass through the β-selection checkpoint proliferate and further differentiate into the CD4/CD8 double-positive (DP) stage. DP cells rearrange the TCRα gene so as to express the complete αβTCR/CD3 complex that is capable of recognizing pMHC. Given that newly generated DP cells express a randomly rearranged αβTCR irrespective of their ligand binding ability, they include harmful cells as well as useless cells in addition to the useful population. DP cells expressing a αβTCR that strongly interacts with self-pMHC are self-reactive and potentially harmful T cells. These cells receive strong αβTCR signals upon the recognition of the self-pMHC complex in the thymus and are eliminated by apoptosis. This process is called “negative selection”. In addition, DP cells that fail to produce pMHC-reactive αβTCR are also destined to die, a process referred to “null selection” or “death by neglect.” DP cells with αβTCR that interact with self-pMHC with a relatively weak affinity are potentially immunocompetent T cells, and receive moderate αβTCR signals that induce differentiation into CD4 single-positive (SP) or CD8SP cells. This process is called “positive selection” [46]. This positive selection occurs in the thymic cortical microenvironment, where cortical thymic epithelial cells (cTECs) produce a set of self-peptides that confer a low-affinity binding on the TCR. The positively selected CD4SP and CD8SP cells then migrate from the cortex into the medulla of the thymus, where SP cells are screened for self-reactivity against the pMHC displayed by medullary thymic epithelial cells (mTECs). Strong αβTCR interaction with pMHC in the medulla leads to negative selection or differentiation into regulatory T cells, ensuring the self-tolerance of T cells [1, 47]. Thus, precise regulation of the αβTCR signal is critical for the generation of a diverse, useful, and yet self-tolerant T cell population.

Unlike the pre-TCR signal during the course of β-selection, the αβTCR signal for positive and negative selection depends on Zap70 [35]. Mice lacking Zap70 but not Syk exhibit a complete loss of αβTCR signaling and T cell differentiation arrest at the DP stage [48, 49]. Consistent with this, disruption of positive selection has also been observed in mice deficient for Lck [50], RhoH [40, 41], or Grb2 [51], indicating that the αβTCR-Lck-Zap70 axis plays a non-redundant role in αβT cell development.

Studies of animals with αβTCR signaling mutations have indicated that properly controlled αβTCR signal strength is required for positive selection of immunocompetent αβT cells. The Zap70 W163C mutation in SKG mice**,** which changes the threshold of the TCR signal needed for positive and negative selection, leads to positive selection of self-reactive T cells and autoimmunity in mice [52]. Themis is a putative adaptor protein that recruits Shp1 to the Lat signalosome during positive selection [2, 53–57]. It is still controversial whether Themis activates Shp1 to tune down the αβTCR signal strength and thus rescue immunocompetent αβT cells from deletion or inhibits SHP1 activity so as to tune up the αβTCR signal and thereby ensure positive selection of αβT cells expressing low-affinity αβTCR [58–60]. Regardless, many studies with Themis-deficient mice have shown that this protein is required for positive selection [53–57]. In addition, serine/threonine-protein kinase D2 (PKD2) and PKD3 are reported to phosphorylate Shp1 and control its function upon αβTCR signaling. Mice with a deficiency of PKD2/3 or with unphosphorylated mutation in Shp1 exhibit abrogated positive selection of CD4SP cells [61]. Thus, precise regulation of αβTCR signal strength by protein phosphorylation is essential for thymic αβT cell development.

Downstream regulators of αβTCR signaling have also been reported to critically control the positive selection of αβT cells. Tespa1, a protein localized to the endoplasmic reticulum membrane, interacts with IP3R1, which activity facilitates calcium ion influx and subsequent MAPK activation [62]. TRAF3-interacting protein 3 (TRAF3IP3) recruits mitogen/extracellular signal-regulated kinase (MEK) and Braf to the Golgi, a process which is required for ERK activation [63].

## γδTCR signaling in γδT cell development

### γδ-selection

γδT cells emerge from DN thymocytes, as the rearrangement of the TCRγ and δ chains occurs in the DN stages [64]. γδ precursor cells, which have TCRγ and δ rearranged prior to TCRβ recombination, express γδTCR/CD3 complex on the plasma membrane, where γδTCR self-oligomerizes, like the pre-TCR, and initiates intracellular signaling pathways [11]. This γδTCR signal induces the process referred to as “γδ-selection,” which confirms the generation of functional TCRγδ chains, making the cell recognize that “I am a γδT cell” [65].

The γδ-selection signal triggers the differentiation from CD5^−^ CD24^high^ γδ precursor cells to CD5^+^ CD24^low^ γδT-committed cells [66]. The transition from CD5^−^ to CD5^+^ γδT cells is markedly impaired in Syk-deficient mice, while Zap70-deficient mice display normal differentiation of CD5^+^ γδT cells. Zap70/Syk doubly-deficient mice exhibit a complete arrest of γδT cell differentiation at the CD5^−^ precursor stage [67]. Thus, γδ-selection is mainly dependent on the Syk-mediated signal, and Zap70 plays only a minor and redundant role in this process. This mechanism is quite analogous to that of β-selection. One critical target of Syk in γδ-selection signal is the Lat signalosome, as Lat-deficient mice exhibit complete inhibition of γδ-selection and a total lack of mature γδT cells [66, 67].

γδ precursor cells from Syk/Zap70-deficient mice or Lat-deficient mice are indistinguishable from αβT lineage cells by the expression of their cell-surface proteins except for the γδTCR, and still maintain the potential to differentiate into αβT cells. What determines the differentiation fate into the αβT or γδT lineage from the precursor? This question has been addressed by studies using γδTCR transgenic mice. When the γδTCR signal is weakened by a deficiency of either signaling proteins or endogenous ligands for the transgenic γδTCR, the precursor cells gave rise to αβT lineage DP cells at the expense of γδT lineage cells [68, 69]. These results suggest that a stronger signal (likely upon γδTCRligand interaction) leads to the commitment to γδT cells, while a weaker signal (likely by ligand-independent pre-TCR) leads to αβT differentiation. However, experiments using another transgenic mouse strain expressing γδTCR with the same ligand-specificity demonstrated that γδT cells were able to mature in the absence of the ligands [70]. Chien and co-workers employed a tetrameric staining method to identify the ligand-specific γδT cell population in order to examine the significance of endogenous γδTCR ligands in non-transgenic mice. The results clearly showed that the number of the ligand-specific γδT cells was comparable between the ligand-sufficient and -deficient mice, suggesting that the majority of γδT cells have not encountered ligands during thymic differentiation [11]. The authors also provided evidence that some γδTCRs can signal ligand-independently [71]. These observations evidently contradict the previous model that the γδT lineage commitment requires γδTCR ligand interaction. Given that polyclonal γδT cells reactive to certain exogenous ligands differentiate and functionally mature in the thymus, it is likely that the observations in certain γδTCR transgenic mouse lines do not reflect the majority of γδT cells with polyclonal γδTCRs.

**Fig. 2:**
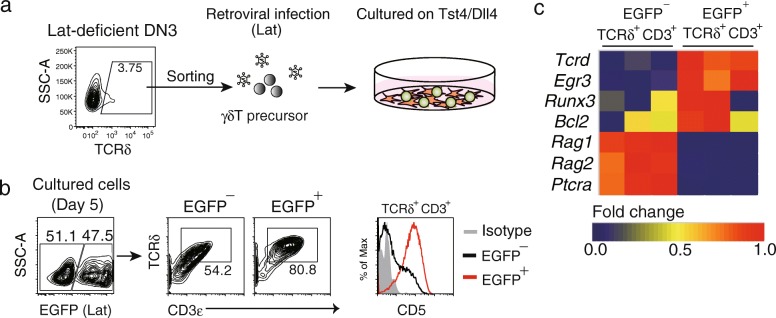
The impact of γδ-selection on αβT/γδT cell fate decision. **a** Scheme of experimental procedure. γδT cells from thymus of Lat-deficient mice were sorted by FACS, infected with retroviruses expressing Lat along with EGFP, and cultured on Tst4/Dll4 stromal cells [103] for 5 days. **b** Expression of TCRδ chain and CD5 in the cultured cells with or without Lat. **c** EGFP^+^ (Lat-transduced) cells and EGFP^−^ (non-transduced) were subjected to qRT-PCR to measure the mRNA expression levels of indicated genes. The heat-map indicates the relative gene expression

To examine the impact of γδ-selection signal on αβT/γδT differentiation, we utilized Lat-deficient mice, in that γδT cell differentiation is arrested at the CD5^−^ precursor stage. γδTCR^+^ precursor cells were purified from adult Lat-deficient mice, infected with retroviruses expressing Lat, and cultured on stromal cell monolayers (Fig. 2a). This experiment allows direct evaluation of the cell phenotype before and after γδ-selection under a ligand-free condition. Compared to non-transduced control cells, Lat-expressing γδT cells displayed a marked induction of the surface expression of CD5 (Fig. 2b), as well as mRNA expression of γδT cell signature genes (*Tcrd*, *Egr3*, *Runx3*, and *Bcl-2*), and complete abrogation of transcription of genes associated with precursor DN cells and αβT cells (*Rag1*, *Rag2*, and *Ptcra*) (Fig. 2c). These results indicate that the γδTCR signal both drives differentiation toward the γδT lineage and represses differentiation into the αβT lineage in a ligand-independent manner.

Taken together, although the mechanisms are still elusive (and debated) by which the pre-TCR and γδTCR direct the differentiation processes into the αβT and γδT lineages, respectively, it is likely that γδ-selection, at least in the majority of naturally generated γδT cells, is not contingent on cognate γδTCR ligand in the thymus.

### γδTCR signal strength determines γδT17/γδT1 differentiation

During the development of both the αβT and γδT lineages, the expression of Syk and Zap70 is inversely regulated: Syk is highly expressed in the early stages (DN1-3 and γδ precursor) and downregulated thereafter, while Zap70 is expressed in the later stages (after β-selection or γδ-selection) [72]. γδT cells that have passed through γδ-selection express high levels of Zap70 as well as γδTCR/CD3 complexes and can respond to endogenous ligands if they are provided in the thymus. It is currently recognized that unlike αβT cells, γδT cells do not undergo ligand-driven positive selection or clonal deletion in the thymus. Several studies have suggested that the γδTCR ligand interaction in the thymus instead controls the effector function of γδT cells.

Using tetrameric staining of a γδT cell population that is reactive to the non-classical MHC class I molecules T10 and T22, Chien’s group found that antigen-naïve γδT cells that developed in the absence of the ligands preferentially produced IL-17, whereas antigen-experienced γδT cells that developed in the presence of the ligands predominantly produced IFNγ [11]. This study first suggested the idea that a ligand-induced strong γδTCR signal and a weak γδTCR signal induce γδT1 and γδT17 cells, respectively. A recent study with newly generated T10/T22-deficient mice reported essentially the same results, supporting this “signal strength model” [73]. This model has been further supported by other studies. The thymic maturation and effector differentiation of Vγ5Vδ1 γδT cells require Skint1 (and likely other Skint family proteins), a putative costimulatory protein for the Vγ5Vδ1 TCR [74–76]. In the absence of Skint1, Vγ5Vδ1 γδT cells are misdirected to a γδΤ17 cell phenotype at the expense of the γδΤ1 cell phenotype [77]. Furthermore, γδT1 cell development also requires costimulation via CD27, a TNF receptor superfamily protein expressed in γδT1 cells, but not γδT17 cells [78]. More recently, Pennington’s group identified thymic bipotent γδT cells (CD24^lo^ CD44^lo^ CD45RB^lo^) which can give rise to both γδT17 cells and γδT1 cells. In fetal thymus organ culture, the development of γδT17 cells was inhibited by strong TCR signals induced by stimulation with anti-TCRδ or anti-CD3ε antibodies, but these effects were abrogated by pharmacological inhibition of the MEK/ERK pathway [79]. These data provide direct evidence in support of the idea that γδTCR signal strength is a critical determinant of γδT cell effector function.

At the transcriptional level, the strong γδTCR signal induces the expression of γδT1-associated transcriptional regulators, such as Egr2, Egr3, and Id3, resulting in a γδT1 cell fate [64]. Id3 inhibits adoption of γδT17 cell fate by inhibiting the transcriptional regulation mediated by HEB (encoded by Tcf12) [80]. HEB can directly bind upstream of the transcriptional start sites of Sox4 and Sox13 [81] to promote their expression. These γδT17-associated transcriptional factors are required for expression of the essential transcriptional factor RORγt (encoded by Rorc) and the signaling protein Blk [82]. Considering these facts, the TCR signal strength model clearly demonstrates the mechanisms by which the TCR signal controls the effector function of γδT cells.

However, a series of studies has demonstrated the impact of the genetic ablation of TCR signaling molecules on γδT cell effector function, challenging the idea that γδTCR signal strength alone determines the γδT17/γδT1 differentiation fate. Zap70 W163C mutant mice exhibit a complete loss of Vγ6^+^ γδT17 cell development but have normal development of γδT1 cells, while TCR signals are dampened in these mice [83]. Another study by Silva-Santos and co-workers showed that mice haploinsufficient for CD3δ and CD3γ (CD3DH), which had lower cell-surface expression of the γδTCR/CD3 complexes and impaired γδTCR signaling, displayed a marked reduction of the thymic development of Vγ6^+^ γδT17 cells as well as γδT1 cells but not of Vγ4^+^ γδT17 cells, indicating that the γδT17 subsets require distinct γδTCR signal strength for their development [84]. Although αβT cell development and αβTCR signal transduction were unaffected in CD3DH mice, this mouse strain is the only animal model thus far in which the specific inhibition of γδTCR signaling has been demonstrated. It remains unclear why γδT cells are specifically affected in CD3DH mice, but it is likely that the distinct composition of the TCR-CD3 complexes αβT and γδT cells accounts for the unique phenotype of CD3DH mice. In this context, it should be noted that mice with the CD3ε C80G mutation, which is unable to induce conformational changes in TCR, also exhibit impaired γδT17 cell development but normal γδT1 cell development [85].

### Syk is required for γδT17 differentiation

Recently, we reported a new regulatory mechanism by which the γδTCR-proximal kinases Syk and Zap70 differentially control γδT17 induction [67]. Syk-deficient mice exhibit complete loss of γδT17 cells (both the Vγ4^+^ and Vγ6^+^ subsets) in the thymus. Notably, forced expression of Zap70 in Syk-deficient T-progenitor cells failed to restore the γδT17 cell generation, suggesting a non-redundant role of Syk in γδT17 differentiation. As Syk- but not Zap70-deficient γδT cells display a significant reduction of the Akt phosphorylation induced by γδTCR stimulation, it is indicated that that Syk mediates the γδTCR-induced activation of the PI3K-Akt pathway. PI3K-deficient mice (p110γ^−/−^ p110δ^−/−^) exhibit complete inhibition of γδT17 cell development but are unaffected in terms of γδ-selection (CD5 upregulation) or γδT1 development. Inhibition of PI3K was shown to reduce the expression of γδT17-associated transcription factors (Rorc, Sox13, and Sox4), suggesting crucial role for the PI3K-Akt pathway in inducing the γδT17 differentiation program. In agreement with this, a previous report demonstrated that kinase-inactive PI3Kδ or PI3Kγ-deficient mice exhibit a marked reduction in the peripheral γδT17 cell number and amelioration of γδT17-dependent inflammation [86]. The PI3K–Akt pathway is also required for the differentiation of IL-17-producing αβT (Th17) cells [87], suggesting that this signaling pathway is a common regulatory mechanism shared by αβT and γδT lineages for inducing IL-17-producing proinflammatory subsets.

γδTCR-induced activation of the PI3K-Akt pathway depends on Syk but not Lat, indicating that Syk drives the PI3K-Akt pathway for inducing γδT17 differentiation in addition to the Lat-dependent mainstream signaling pathway that induces γδ-selection [67]. It is unclear whether Syk activates the PI3K/Akt pathway in γδT cells through direct interaction or in an indirect manner. A previous study reported that Rasgrp1-deficient mice display a γδT cell effector phenotype similar to that of PI3K-deficient mice (i.e., a loss of γδT17 cells and increase of γδT1 cells) [88]. Since Rasgrp1 can function as an upstream activator of the PI3K/Akt pathway in αβTCR signaling [89], it is likely that Rasgrp1 relays signals from γδTCR to PI3K to induce γδT17 differentiation.

Preferential loss of γδT17 cells was also reported in mice deficient for Blk, a Src family kinase expressed in γδT cells as well as B cells, although its function in γδTCR signal transduction is unknown [82].

### Zap70 controls certain γδT cell subsets

We have also demonstrated the role of Zap70 in the thymic differentiation of γδT cells [67]. Zap70-deficient mice display a marked reduction of Vγ6^+^ cells, the majority of which are γδT17 but are unaffected in terms of the development of other γδT cells, including the Vγ1^+^ as well as Vγ4^+^ subsets. Indeed, the expression level of the Zap70 protein was highest in the Vγ6^+^ subset among the γδT cells. As the CD5 expression was lower in the Zap70-deficient Vγ6^+^ cells than control cells, Zap70 is likely required for thymic maturation of Vγ6^+^ cells. In our experiments, Zap70-deficient mice had normal thymic differentiation of Vγ4^+^ cells, including the γδT17 subset, which contradicts the previous report in which a hypomorphic Zap70 mutation caused a reduction of thymic Vγ4^+^ γδT17 cells [83]. This discrepancy may be due to the different mice used in the two studies (Hayday’s group used hypomorphic Zap70 mutant mice on a BALB/c background, whereas we used complete Zap70-deficient mice on a C57BL/6 background). In addition, Zap70-deficient mice displayed a significant reduction in peripheral Vγ4^+^ cells, which included both the γδT17 and γδT1 subsets, but had unimpaired Vγ1^+^ cells. Thus, in contrast to its essential role in αβT cell development, the requirement of Zap70 is limited to the thymic maturation of Vγ6^+^ cells and peripheral maintenance of Vγ4^+^ cells.

Our findings on the different roles of Zap70 and Syk might provide a new clue to understand the mechanisms of γδTCR signaling and γδT cell development. Zap70 is required for αβTCR signaling and γδTCR signaling in certain γδT cell subsets. In αβT cells, the activation of Zap70 is dependent on Lck, which is coupled with CD4 or CD8 coreceptors that bind to pMHC on the surface of antigen-presenting cells [90]. Thus, it is suggested that Lck-Zap70 is a signaling axis that is specialized in antigen recognition achieved by cell-cell contact; although in the case of γδT cells, it remains unclear how Zap70 is activated despite the lack of CD4 and CD8 expression. In contrast, Syk is associated with a wide range of immunoreceptors, including pre-TCR, γδTCR, BCR, and FcR [37]. Because Syk is capable of phosphorylating ITAMs and downstream targets independently of Src family kinases such as Lck [91], these receptors can be activated ligand-independently or upon binding to a variety of soluble as well as cell-surface antigens. Thus, the utilization of Syk or Zap70 in immunoreceptor signaling may dictate how the receptor recognizes antigen. Indeed, the expression of Syk in place of Zap70 rendered αβT cells capable of responding to soluble anti-CD3 antibody stimulation, while normal αβT cells only responded to multimerized anti-CD3 antibodies that mimic the interaction with cell-surface pMHC [92]. These findings prompted us to hypothesize that the mode of antigen recognition used by lymphocytes might be determined not only by their receptor per se, but also by distinct usage of Syk family kinases. Based on this concept, we predict that there are endogenous cell-surface γδTCR ligands required for thymic maturation of Vγ6^+^ cells, as well as the peripheral maintenance of Vγ4^+^ cells, and that Vγ1^+^ cells do not require cell-surface γδTCR ligands for their development and/or maintenance.

### γδTCR-independent and -dependent processes for γδT17 induction

A recent report elegantly demonstrated that γδT17 cells arise from a progenitor that is distinct from the other γδT cell subsets [93]. It was reported that fetal-origin, intrathymic progenitors expressing high levels of Sox13 were identified in a population previously categorized as DN1d (CD44^+^CD25^−^c-kit^−^CD24^hi^) thymocytes. These Sox13^+^ progenitors preferentially gave rise to γδT17 cells in the reconstituted fetal thymus, whereas other progenitors within the DN2 population did not. Most importantly, the Sox13^+^ progenitors were detectable and their γδT17 lineage programs were intact in TCRδ-deficient or Rag-deficient mice, indicating that the γδT17 lineage fate is “prewired” by a cell-intrinsic, γδTCR-independent mechanism. A previous report, however, showed that γδT17 cells can develop from the DN2 stage (CD44^+^CD25^+^c-kit^hi^) when co-cultured on a monolayer of Notch ligand-expressing stromal cells [15]. There may thus be a need to redefine the differentiation stages and progenitor-descendant relationships in γδT cell development.

**Fig. 3:**
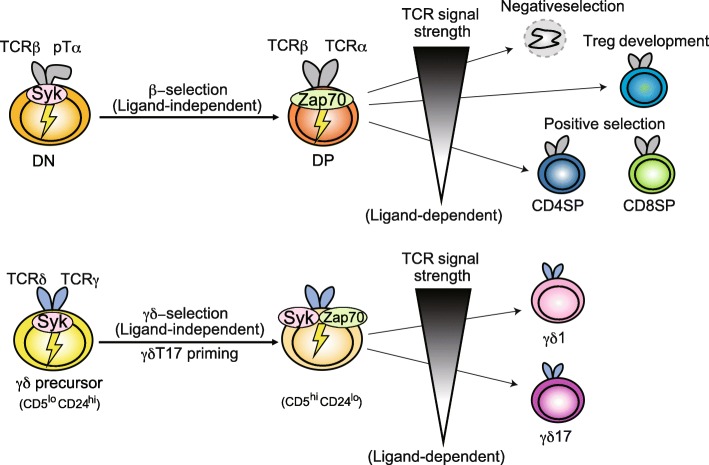
Distinct requirement of Syk family kinases for thymic development of αβT/γδT cells

Figure 3 summarizes the differentiation processes of γδT cells as well as αβT cells, highlighting the differences in the requirement of αβ/γδTCR signals and Syk family kinases. The early steps of differentiation, i.e., β-selection for the αβT cell lineage and γδ-selection for the γδT cell lineage, are driven by ligand-independent pre-TCR or γδTCR signaling, which serves as a checkpoint for the cells expressing a functional TCRβ chain or γδTCR chain, respectively. These ligand-independent receptor signals are initiated by Syk, which is expressed in DN thymocytes, including γδT precursors. In the γδT cell lineage, Syk-mediated γδTCR signal is also required for the priming of γδT17 cell differentiation via the activation of the PI3K pathway. During both β-selection and γδ-selection, the expression of Syk and Zap70 is inversely regulated: Syk is downregulated while Zap70 is upregulated upon pre-TCR or γδTCR signaling. Therefore, the later step in αβT lineage differentiation depends on Zap70-mediated αβTCR signaling, which allows DP thymocytes to recognize pMHC on the surface of TECs in order to be positively or negatively selected according to the strength of the αβTCR-pMHC interaction. In contrast, Zap70-mediated γδTCR signaling in response to endogenous ligands determines the effector function of γδT cells: a strong signal induces γδT1, while a weak/no signal induces γδT17.

## Control of γδT17 cells by non-γδTCR signals

It has also been reported that the development of γδT17 cells is regulated by non-TCR factors, such as Notch ligands and cytokines. γδT17 cells highly express Notch1 and, upon the binding with its ligand Dll4 that is expressed by cTECs, induce the expression of the transcriptional repressor Hes1. Genetic ablation of Hes1 impairs the development of γδT17 cells but not γδT1 cells, indicating the critical role of the Notch-Hes1 pathway in γδT17 differentiation [94]. TGF-β1 is also required for the optimum generation of γδT17 in the thymus [95]. IL-7 induces the expansion of γδT17 cells in the thymus and at the periphery [96]. A recent study showed that the production of IL-7 in the thymus is negatively controlled by Aire, a transcription regulator expressed in mTECs. Mice lacking Aire exhibit an increased production of IL-7 and thereby selective overproduction of γδT17 cells, which at least partly accounts for the inflammatory disorders in these mice [97]. Other studies have also demonstrated that TECs critically control the differentiation of γδT17 cells. In the thymus of mutant mice lacking mature cTECs, the frequency of γδT17 cells is greatly increased. Among these γδT17 cells, the Vγ6 subset was increased, whereas the Vγ4 subset was decreased [98]. A similar increase of Vγ6 γδT17 cells in the thymus was observed in mice deficient for mTORC1/Raptor [99]. Deletion of NIK, a kinase required for NF-κB activation and mTEC development, caused a marked reduction of both Vγ5 γδT cells and γδT17 cells [100]. Thus, the normal development of TECs critically contributes to the repertoire formation of γδT17 cells, although its mechanism remains unclear.

Haas et al. reported that γδT17 cells do not develop in a mouse model that allows Rag1 expression only at the adult stage [101]. In a model drug-induced conditional γδT cell depletion, γδT17 cells recovered very inefficiently, while γδT1 cells readily recovered after depletion [14]. In addition, transplantation of bone marrow cells into lethally irradiated mice failed to reconstitute the thymic development of γδT17 cells, whereas mice reconstituted with fetal liver cells were capable of generating Vγ4 γδT17 cells in the thymus [67, 101]. Therefore, the thymic development of γδT17 cells requires fetal liver-derived progenitors and Vγ6 γδT17 cells additionally require a fetal thymic microenvironment for their differentiation and maturation. Under inflammatory conditions, however, it was shown that bone marrow-derived naïve Vγ4 γδT cells can be induced to produce IL-17 in peripheral lymphoid tissues, a result in which IL-23 and IL-1β are critically implicated [102].

## Conclusion

Although γδT cells are one of the three types of antigen receptor-expressing lymphocytes conserved among all vertebrates, their functions and developmental mechanisms have long been enigmatic compared with those of αβT and B cells. As discussed in this review, a series of recent studies has unveiled the various roles of γδT cells under both physiological and pathological conditions, along with the regulatory mechanisms for the differentiation of proinflammatory γδT cells. In particular, it has been demonstrated that γδT cells have certain unique features in the TCR/CD3 complex and its downstream signaling pathways that dictate their maturation and effector function. The remaining issues to be resolved include the function of γδTCR-specific signaling proteins (such as Blk), full characterization of the γδT cell subsets and their precursor-product relationships, and identification of the endogenous γδTCR ligands that control thymic γδT cell differentiation. From a therapeutic perspective, it is critical to determine whether manipulation of γδTCR signaling can treat and/or protect against infection, autoimmunity, and cancer.

## Abbreviations

Blk: B lymphoid kinase
Lat: Linker for activation of T cells
Lck: Lymphocyte protein tyrosine kinase
PLCγ1: Phospholipase C, gamma 1
Shp1: Src homology region 2 domain-containing phosphatase 1
Skint1: Selection and upkeep of intraepithelial T cells 1
Sox13: SRY (sex determining region Y)-box 13
Syk: Spleen tyrosine kinase
Zap70: Zeta-associated protein of 70 kDa
